# Developing a Virtual Model of the Rhesus Macaque Inner Ear

**DOI:** 10.3390/bioengineering11111158

**Published:** 2024-11-18

**Authors:** Cayman Matson, Nicholas Castle, Chenkai Dai

**Affiliations:** 1 Stephenson School of Biomedical Engineering, University of Oklahoma, Norman, OK 73019, USA; cayman.t.matson-1@ou.edu; 2 Aerospace and Mechanical Engineering, University of Oklahoma, Norman, OK 73019, USA; ncastle@ou.edu; 3 Biomedical Engineering Department, Johns Hopkins University, Baltimore, MD 21218, USA

**Keywords:** inner ear, finite element, computational model, rhesus macaque

## Abstract

A virtual model of the rhesus macaque inner ear was created in the present study. Rhesus macaques have been valuable in cochlear research; however, their high cost prompts a need for alternative methods. Finite Element (FE) analysis offers a promising solution by enabling detailed simulations of the inner ear. This study employs FE analysis to create a virtual model of the rhesus macaque’s inner ear, reconstructed from MRI scans, to explore how cochlear implants (CIs) impact residual hearing loss. Harmonic-acoustic simulations of sound wave transmission indicate that CIs have minor effects on the displacement of the basilar membrane and thus minimally impact residual hearing loss post-implantation, but stiffening of the round window membrane worsens this effect. While the rhesus macaque FE model presented in this study shows some promise, its potential applications will require further validation through additional simulations and experimental studies.

## 1. Introduction

### 1.1. Cochlear Implants and Residual Hearing

Across the globe, sensorineural hearing loss affects up to 5.2% of individuals [1]. For those with sensorineural hearing loss, traditional hearing aids are ineffective due to dysfunctions in the structures of the ear. Cochlear implants (CI) bypass the typical auditory pathway to directly stimulate the auditory nerve via electrical impulses. CI surgery is a widely utilized and effective treatment for severe hearing sensorineural loss, with over one million implants performed globally [2].

Despite their successes, CIs are not without their flaws. Individuals with hearing loss often do not suffer from complete deafness. The remaining hearing ability after hearing loss is dubbed residual hearing. However, about half of adults with CIs report the loss of any residual hearing that was present before the surgery [3]. As residual hearing improves hearing quality in patients with CIs, the preservation of residual hearing has a become a priority for researchers [3]. The mechanisms underlying residual hearing loss following CI surgery remain uncertain. Potential contributing factors include trauma during surgery, postoperative tissue stiffening, fibrosis within the inner ear, and style of CI [4,5,6,7]. Previous in vivo experiments have been limited by low sample sizes, and ex vivo studies are limited in that it is difficult to predict how CIs will affect residual hearing in nonliving subjects [7]. Only one prior study has been conducted on residual hearing loss due to CIs in silico, which utilized a human model instead of the rhesus macaque [6].

This study is novel in that it seeks to explore how CIs affect residual hearing loss in both atraumatic insertions (with no fibrotic tissue stiffening) and in traumatic insertions (wherein the round window membrane scarifies after insertion) in the rhesus ear. Given the prevalence of cochlear implants and their associated challenges, further investigation into CIs, especially in animal models used for otological research, is crucial to fully understand their impact on ear mechanics.

### 1.2. The Rhesus Macaque as an Animal Model

Animal testing remains a cornerstone of otological research, particularly in the study of cochlear implants. Nonhuman primates are often considered the gold standard for inner ear research due to their close physiological and neurological similarities to humans. The rhesus macaque (*Macaca mulatta*) is an exemplary model organism in this context, as its cochlea shares comparable structural features with the human cochlea, including the same number of turns and similar proportions [8,9]. Neurologically, the auditory processing functions in rhesus macaques are also analogous to those in humans [10,11]. Previous studies have demonstrated the suitability of rhesus macaques for cochlear implant research, underscoring their value in evaluating implant viability [8,12]. Animal models cannot perfectly stand in for humans, as the rhesus ear is not exactly the same as the human ear, but they are as a necessary precursor to any human trials.

However, the high cost of housing and maintaining these animals poses a significant barrier to many researchers. To address this challenge, there is a growing need for cost-effective alternatives, such as virtual models. These virtual models offer a flexible platform for experimentation, allowing researchers to modify parameters, test different scenarios, and refine their approach based on simulated results. By conducting initial experiments in silico, researchers can optimize cochlear implant designs and experimental protocols, thereby potentially reducing the number of live subjects required. For instance, a virtual model of the rhesus inner ear could be used to iteratively test and refine cochlear implant designs, focusing in vivo experiments only on the most promising solutions. Developing a virtual model of the rhesus macaque’s inner ear can serve as a preliminary step before in vivo studies. This approach not only saves time and resources but also minimizes the need for animal testing.

### 1.3. Computational Modeling and Previous Ear Models

Creating an accurate virtual model of the ear is a complex challenge, but Finite Element (FE) analysis offers a powerful tool for this task. FE analysis is a technique used in mathematical and engineering modeling to solve complex systems by breaking them down into smaller, manageable parts called finite elements. This approach involves subdividing a large system into discrete elements, often in the shape of tetrahedral or hexahedral cells, which together form the model’s mesh. Each element is assigned specific mechanical properties and boundary conditions to accurately simulate the behavior of the tissues [13]. The model is then tested against in vivo data and refined to ensure realistic results. Once validated, FE models enable highly precise and rapid simulations for a variety of experiments in silico.

FE analysis has been widely used for modeling the ear in various species. Previous studies have used chinchilla models to characterize the functions of the middle ear in both the chinchilla [14] and the gerbil [15]. Our laboratory has previously applied FE techniques to develop a model of the chinchilla to investigate the effects of CI implantation angles [16]. Similarly, successful models of the human ear have been created to analyze residual hearing loss and ear function [6,17].

However, there has been little work carried out to model the ear function of the rhesus macaque. To our knowledge, our laboratory’s previous model of the rhesus vestibulo-ocular reflex (VOR) response to electrical stimulation is the only model to explore rhesus otology [18]. However, this model lacked detailed reconstructions of the membranous labyrinth, lymphatic system, and cochlear anatomy. This study seeks to improve on our previous investigations by modeling high-resolution reconstructions of the inner membranes of the rhesus cochlea.

### 1.4. Experimental Focus of the Study

This study presents the development of a virtual model of the rhesus macaque’s inner ear, aimed at investigating the impact of cochlear implants (CIs) on basilar membrane displacement. The model was reconstructed from high-resolution µMRI scans of a deceased macaque and includes the entire inner ear structure: the cochlea, vestibule, and semicircular canals. The cochlea is responsible for converting acoustic waves to neural impulses. The vestibule contains the utricle and saccule, which control the body’s sense of equilibrium and balance. The semicircular canals assist in sensing the rotational position and movement of the head. Together, these structures make up the membranous labyrinth. The membranous labyrinth is filled with plasma-like lymphatic fluids, either endolymph or perilymph, which assist in sound transmission and support the vestibular organs.

The model enables simulations of sound wave transmission through the oval window and into the cochlea, allowing for the measurement of basilar membrane displacement. This displacement is crucial for sound perception and is linked to tonotopic mapping, where specific frequencies correspond to distinct locations and amplitudes of membrane displacement [19]. By adjusting key parameters in the model—such as the stiffness of the round window, the density of the lymphatic fluids, or the material properties of the implanted electrode—we can analyze how these changes affect basilar membrane displacement. Significant deviations in this displacement may indicate alterations in hearing or the loss of residual hearing ability.

Previous studies have suggested that CI implantation does not significantly affect cochlear wave mechanics, attributing residual hearing loss primarily to post-operative tissue stiffening rather than the presence of the electrode in the scala tympani [6]. This study aims to revisit these findings using a detailed rhesus macaque inner ear model to investigate if cochlear implants and stiffening of the round window membrane negatively affect residual hearing loss.

## 2. Materials and Methods

The model was digitally reconstructed from a high-resolution µMRI scan of a rhesus macaque’s (Macaca mulatta, female, 9.2 kg adult) inner ear. All procedures were conducted in accordance with a protocol approved by the Johns Hopkins Animal Care and Use Committee and The University of Oklahoma Animal Care and Use Committee. Material properties for the various ear structures were sourced from the existing literature and integrated into the model’s geometry. Harmonic acoustic simulations were then conducted to simulate the vibration of the stapes and the resultant displacement of the basilar membrane. The model was iteratively refined to ensure that the simulation results aligned with experimental data. Once optimized, a cochlear implant was incorporated into the simulation to evaluate its impact on ear function.

### 2.1. Model Geometry

**Figure 1:**
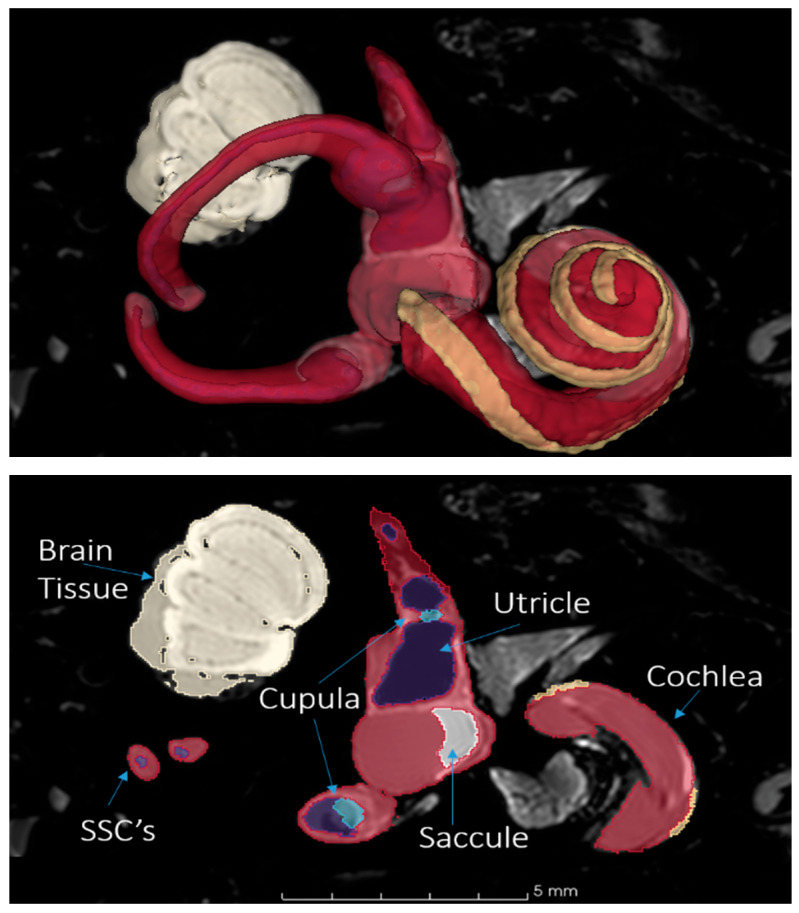
Segmentation of the rhesus inner ear with 3DSlicer from µMRI scan. **Top**: a 3D view of the fully segmented cochlea, vestibule, and semicircular canals (SSCs). **Bottom**: a planar view of the model with structures annotated. Yellow highlights indicate the outer bounds of the scala media. Red highlights indicate the fluid of the perilymph. Blue highlights indicate the fluid of the endolymph.

3DSlicer is an open-source software for reconstructing medical scans into 3D models and is one of the most commonly used tools for image analysis in biomedical research [20]. 3DSlicer (version: 4.11.20200930) was utilized to segment the structures of the inner ear from µMRI datasets of the rhesus macaque. These segmented structures formed the foundation for constructing the 3D geometry of the model (Figure 1). To ensure the highest accuracy, the segments were rigorously cross-checked against existing measurements and anatomical data from the literature.

The model includes reconstructions of the whole of the inner ear. While the primary focus of this study is on the mechanical responses of the basilar membrane within the cochlea, including the entire inner ear provides a comprehensive understanding of how the membranous labyrinth’s geometry influences the system. The interaction between the cochlea and other inner ear structures can impact fluid dynamics in the bony labyrinth [21], and a similar interaction may affect the mechanical responses of the basilar membrane. Therefore, the vestibule and semicircular canals were also included in the simulation.

The cochlea was modeled primarily using data from the µMRI scans, though some sections, such as the cochlear chambers, had to be reconstructed due to less clarity in the imaging. The cochlea is divided into three compartments: the scala tympani, scala media, and scala vestibuli. The scala media, which contains the endolymph and is crucial for this study, is bordered by the Reissner’s membrane above, the basilar membrane below, and the stria vascularis laterally. These membranes form a triangular or wedge-shaped structure [22], attaching to prominent ridges within the cochlea and the osseous spiral lamina. Flat planes were created to represent the membranes, with their width increasing towards the apex. The model’s basilar membrane length of 27 mm aligns closely with the average midline length of 26.3 mm reported for rhesus macaques [23].

Modeling the cochlea’s terminal ends, particularly the apex and base, posed challenges due to their complexity. The apex features the helicotrema, a critical structure where fluid from the scala tympani transitions into the scala vestibuli and spirals back down the cochlea. Accurate modeling of this area was achieved through cross-referencing multiple sources [24,25]. Special care was taken to represent how the osseous spiral lamina terminates in a crescent-shaped ‘hook’ beneath the cochlear duct and how the basilar and Reissner’s membranes form a blind sac.

The vestibule, which contains lymphatic fluid and sensory structures, connects to the cochlea via the reuniting duct. The semicircular canals, filled with fluid and connected to the vestibule, also influence the distribution of sound waves within the inner ear. Including these structures in the simulation ensures that the model performs as realistically as possible, accounting for the complex interactions within the inner ear.

### 2.2. Meshing

**Figure 2:**
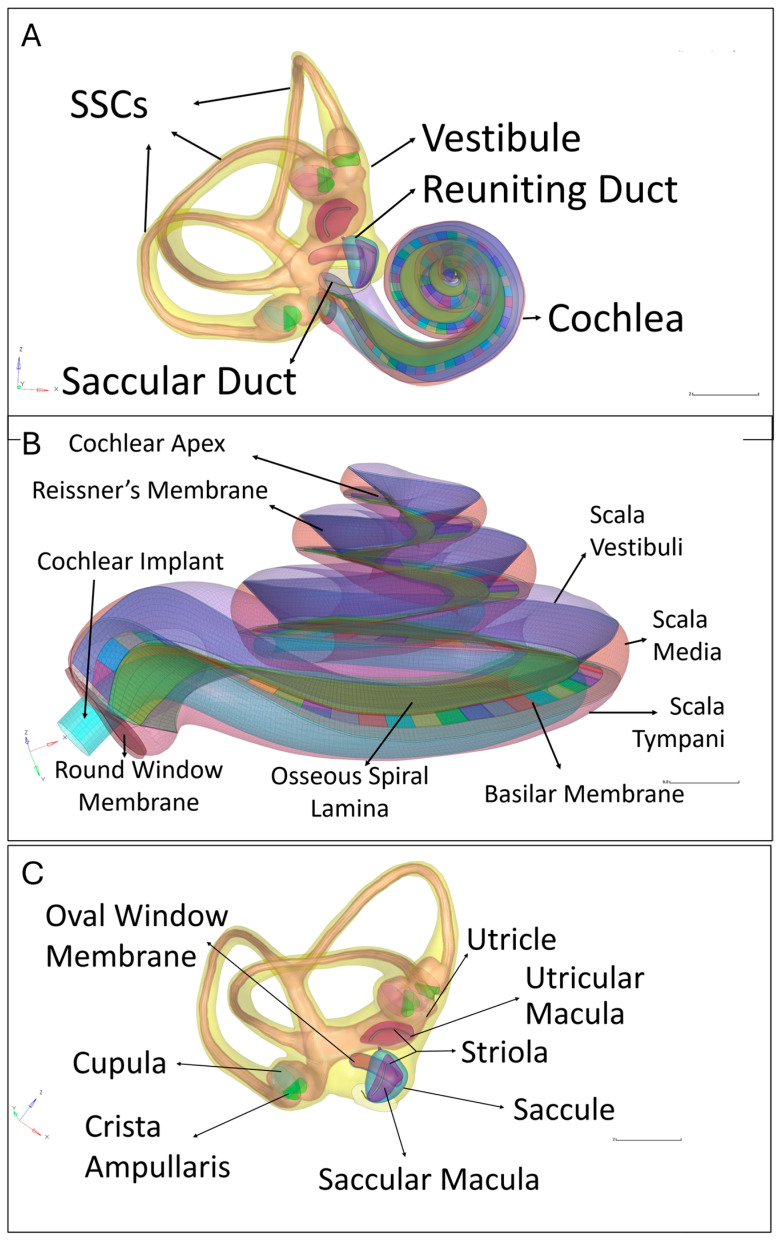
An annotated Hypermesh model of the rhesus inner ear after meshing. (**A**) Full model containing semicircular canals (SSCs), vestibule, and cochlea. (**B**) A zoomed-in view of the cochlea with an electrode placed inside the scala tympani. The basilar membrane is represented as a multicolored strip, subdivided into 82 distinct sections. (**C**) A zoomed-view of the semicircular canals and the vestibule.

The geometry created for the model was imported into SpaceClaim (2021.R1, Ansys Inc., Canonsburg, PA, USA) to lay surface meshes, or skins, on each structure. SpaceClaim, a direct modeling CAD software, was preferentially used over parametric CAD software, such as SolidWorks, as it is better suited for processing organic, complex geometries. Subsequently, Hypermesh (2017.2, Altair Engineering, Troy, MI, USA) was used to combine these skins into a comprehensive mesh model (Figure 2). Hypermesh is an extremely useful tool for preprocessing models in FE analysis due to its robust toolset for generating orderly sets of elements across complex surfaces [26]. Although quadrilateral elements are generally preferred for complex simulations due to their superior accuracy compared to triangular elements, they are more time-consuming to generate [13]. Given the model’s complexity, quadrilateral elements were used preferentially, though some uneven regions required the use of pyramidal elements. The final model comprised 2,657,195 elements and 599,957 nodes.

Hypermesh was also used to construct the electrode of the CI and place it through the round window membrane and within the scala tympani, as is achieved in actual implantations. The electrode placement represents a ‘best-case’ scenario, wherein the electrode was inserted without any trauma to the walls of the cochlea. The dimensions of the electrode were based MED-EL FLEX24™ electrode, scaled down to fit the smaller size of the macaque cochlea [27].

Fluid–structure interactions dictate the vast majority of energy transfer within the ear, as waves must propagate through the lymphatic fluids to create structural deformations in the basilar membrane, which in turn affect the pressure and flow of fluid. For the harmonic-response simulation in ANSYS, elements were classified as either SOLID180 or FLUID30. SOLID180 and FLUID30 are standard element types designed for fluid–structure interaction modeling, with each element featuring 8 nodes and 3 degrees of freedom [13]. SOLID180 was applied to the tissue components, while FLUID30 was used for the lymphatic fluids. The model’s outer boundary, representing the bony layer surrounding the inner ear, was defined as an elastic support layer with a very high elastic modulus to simulate the damping effect of bone. This bony layer is effectively fixed in place, as the temporal bone is much stiffer than any ear tissue and is further dampened by its integration with the skull. Fluid-solid boundaries were established at the interfaces where lymphatic fluids interacted with tissues or the cochlear electrode. As the entirety of the ear is filled with lymphatic fluid, these interfaces include the interiors of the semicircular canals, vestibule, the scalae of the cochlea, and the surface of the electrode.

A mesh convergence analysis (MCA) involves refining the mesh iteratively until the solution stabilizes and becomes independent of mesh size and distribution. This process is essential for optimizing finite element analysis, as finer meshes require more computational resources without necessarily improving accuracy beyond the convergence point. However, due to the intricate nature of manually meshing the inner ear model and its internal structures, MCA was not performed in this study. Instead, this study opted to use a much finer mesh size than previous studies. This model utilizes 2,657,195 elements, compared to approximately 600,000 elements [6] or 500,000 elements [16] used in previous studies. This high mesh density ensures the model is not suffering in accuracy due to mesh coarseness, albeit at the expense of increased computation time.

### 2.3. Mechanical Properties

The mechanical properties of the tissues in the model are crucial for accurately simulating the harmonic acoustic response. These properties guide the program in determining how each material should behave under acoustic stimuli. For this study, the relevant properties vary depending on whether the material is solid or fluid. Solid materials, such as the basilar membrane, require parameters like density, Young’s modulus (elastic modulus), beta damping coefficient, and Poisson’s ratio. In contrast, fluid components like the endolymph necessitate the viscosity and bulk modulus. Due to the lack of comprehensive literature on the mechanical properties of the inner ear, values for these properties were sourced from a variety of references, often relying on human data. Where possible, in vivo measurements of the rhesus ear were selected. In instances where data were missing, in vivo measurements of the human ear were chosen because they, as mentioned previously, are extremely morphologically similar to the rhesus macaque [8,9]. Where neither human nor rhesus in vivo results were available, values were drawn from previous simulated models of the human ear. While the human ear is similar to the rhesus, these substitutions may negatively impact the model’s performance. Future studies would highly benefit from more empirical data on the mechanical properties of the rhesus inner ear and should consider it a priority for improving the model’s rigor. All values and sources are summarized in Table A1 in Appendix A.

The basilar membrane, a key focus of this study, significantly influences the harmonic response within the cochlea. Its density, like most tissues in this model, was set to 1200 kg/m^3^, as per Zhang and Gan (2013) [28]. However, the Poisson’s ratio, damping coefficient, and elastic modulus were experimentally determined. The mechanical properties of the basilar membrane vary along its length, enabling it to resonate at different frequencies at specific locations. To account for these variations, equations were derived to represent these changes. Validating these equations was the most time-consuming aspect of the study, requiring months of iterative testing to ensure the model’s output matched experimental results. Ultimately, first-order polynomial equations were adopted for both the elastic modulus:

[10^(5.7)^ × e^(−0.19×X)^] (Pa) (1)

and the damping coefficient:

[10^(−5.91)^ × e^(0.1×X)^] (unitless) (2)

where *X* represents the distance from the base of the basilar membrane in millimeters.

The mechanical properties of the membranous labyrinth were applied uniformly to the outer walls of the model, as was conducted in similar studies [29]. While this approach is a simplification, the complexity of the various tissues and the limited availability of detailed data necessitate some level of abstraction. Future studies should explore this area further for greater accuracy. Similar to the basilar membrane, the elastic modulus and damping coefficient of the membranous labyrinth were experimentally derived through multiple testing iterations.

The incompressibility of the fluid in the scalae makes the presence of the round window membrane essential, as it allows the fluid to move in a manner similar to compression during expansion and contraction [28]. The mechanical properties of the round window membrane have been documented in similar modeling studies [30]. The oval window membrane, displaced by the stapes’s movement, propagates waves into the cochlea’s interior [31]. However, due to the limited literature on its mechanical properties, it was assumed to share the same properties as the round window membrane due to their similar functions and morphologies. Bounded by the stapedial annular ligament, it allows for a greater degree of flexion, with its properties extensively reported in other studies [32].

Lastly, the electrode’s properties were assumed to be similar to medical-grade silicone [33], with the Pt-Ir wires inside the electrode disregarded due to their relatively small size compared to the silicone sheath. This is a typical simplification for electrode modeling [34].

### 2.4. Simulation and Verification

In the actual ear, vibrations of the stapes and oval window create displacements of the basilar membrane. A given portion of the basilar membrane will experience the greatest displacement when the frequency of the oval window matches its own resonant frequency. In the cochlea, we expect to see a tuning effect wherein the point of resonant frequency shifts away from the base and towards the apex as frequency decreases. There is a mostly exponential relationship between this point and the distance from the base [35]. The Greenwood frequency-position function gives the resonant frequency at a given point along the basilar membrane [36]:

F = A × (10^(ax)^ − k) (3)

where F is the resonant frequency (Hz), A is a constant that varies by species’ basilar membrane length, a and k are set constants, and x is the ratio of the chosen point’s distance from the cochlear apex over the total basilar length. In this model, A is 395, a is 2, and k is 1.

By comparing the initial displacement results of the model to the theoretical predictions from the Greenwood frequency-position function, the model’s mechanical properties can be selectively adjusted until the two align. This process of fine-tuning the model using experimental data is referred to as phenomenological analysis—a widely used technique for validating the accuracy of inner ear models [16,37].

In this study, the basilar membrane’s mechanical properties were modified until the location of maximal simulated displacement matched the estimations provided by the frequency-position function. This phenomenological approach establishes a baseline for the unimplanted cochlea. When a cochlear implant is introduced into the simulation, any deviations from this standard response can be observed, allowing researchers to assess the implant’s impact on cochlear function. Variations in basilar membrane displacement due to the implant may help explain the residual hearing loss experienced by many patients post-implantation.

## 3. Results

### 3.1. Impact on Basilar Membrane Displacement

Displacement was applied to the stapes of the model at varying frequencies and amplitudes to simulate the natural transduction of sound from the middle ear to the cochlea (Table 1). These values were adapted from Gan et al.’s 2007 study of the vibration of the stapes footplate [17]. It should be noted that the study derived these values in vivo experiments with human ears, but unfortunately there is currently no literature on the vibrational patterns of the rhesus stapes with which to replace them.

The displacement of the basilar membrane peaks at the location of its resonant frequency. In the model, the amplitude of this displacement varies, ranging from nanometers (10^−9^ m) at lower frequencies to picometers (10^−12^ m) at higher frequencies. Other studies, such as Chen et al. (2011) [38], have also observed displacement magnitudes generally in the nanometer range.

**Figure 3:**
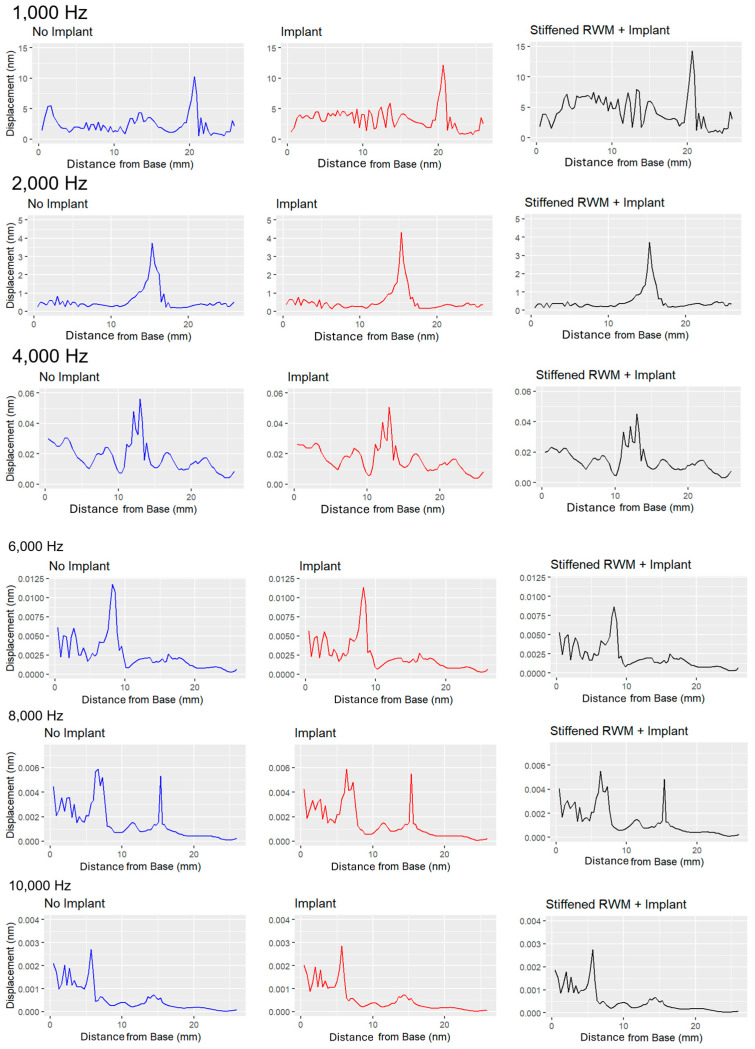
Results of the harmonic-acoustic simulation in Ansys showing the simulated displacement (in nm) of the basilar membrane in response to vibrations of the stapes at six different frequencies in three different electrode implantation scenarios. The location of maximal displacement amplitude indicates the point of resonant frequency for each simulated frequency. As frequency increases, the location of maximal displacement moves closer to the base of the cochlea. **Left**: Unnormalized displacement of unimplanted, healthy cochlea, **Center**: Unnormalized displacement of cochlea with electrode implanted into scala tympani. **Right**: Unnormalized displacement of cochlea with electrode implanted and a stiffened round window membrane.

**Figure 4:**
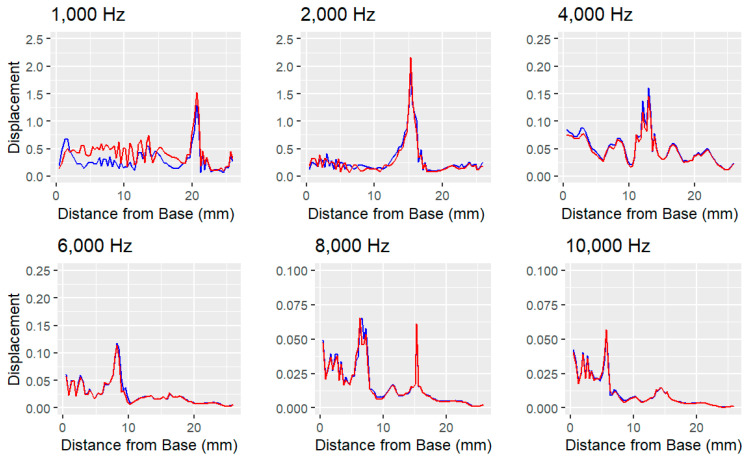
Comparison of the harmonic-acoustic simulation showing the simulated normalized displacement (unitless) of the unimplanted cochlea (blue) and implanted cochlea (red). Membrane displacement was normalized by the displacement of the stapes for each respective frequency. The pattern of displacement is closely mirrored at high frequencies, but it separates at low frequencies.

Figure 3 and Figure 4 show significantly higher noise levels at lower frequencies. In the implanted model, this noise increases, suggesting that the cochlear implant introduces unintended displacements in other regions of the basilar membrane. This interference could negatively impact hearing at lower frequencies, particularly within the critical range of human speech (300 to 4000 Hz) [39]. Despite the presence of the implant, the location of maximum displacement remains consistent between the implanted and unimplanted models.

**Figure 5:**
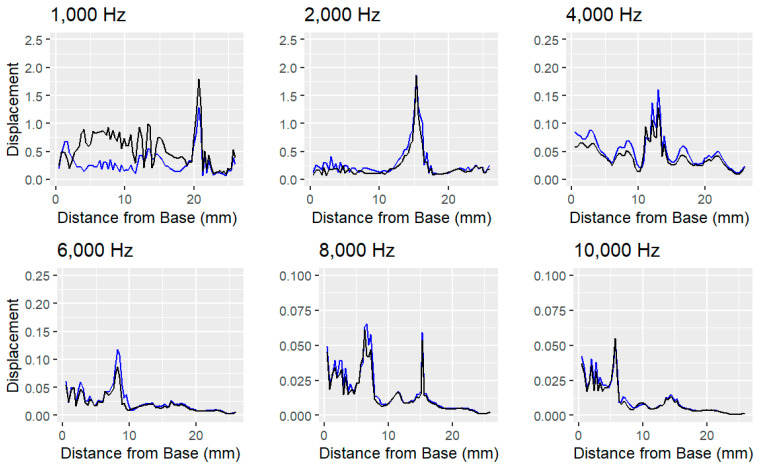
Results of the harmonic-acoustic simulation in Ansys showing the simulated normalized displacement (unitless) of the unimplanted cochlea (blue) and implanted cochlea with a stiffened round window membrane (black). Membrane displacement was normalized by the displacement of the stapes for each respective frequency. Displacement is further distorted in the stiffened and implanted model, especially at low frequencies.

However, when the elastic modulus of the round window membrane was increased tenfold in the implanted model, as carried out by Lim et al. (2020) [6], the differences became more pronounced (Figure 5). As can be seen in Table 2, root mean square error between the displacement of the healthy, unimplanted ear and the stiffened ear is roughly double that of the implanted ear’s error. Noise continued to rise at low frequencies, implying that hearing could become further impaired. Interestingly, the stiffened model showed greater peak displacements at low frequencies but reduced peak displacements at higher frequencies.

### 3.2. Verification

**Figure 6:**
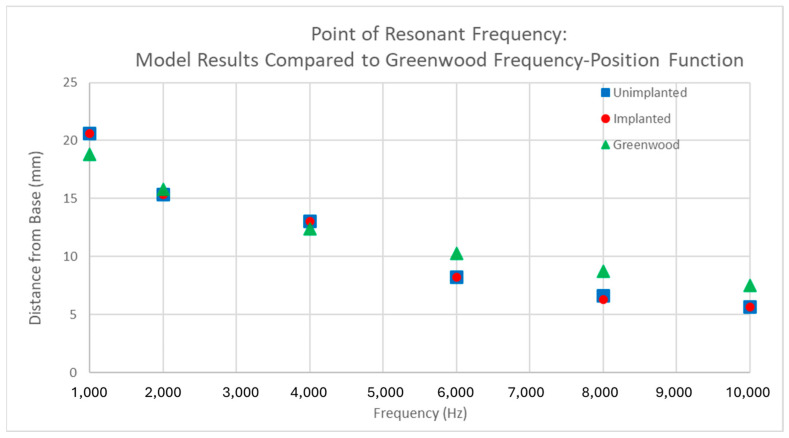
Point of resonant frequency on basilar membrane at different frequencies. The Greenwood results are the expected values from the Greenwood frequency–position function. The implanted and unimplanted values are the locations of peak displacement taken from the simulation output. The implanted and implanted with stiffened RWM had the same location values, so the latter was left out on the graph for brevity.

The model was validated by comparing the point of resonant frequency across a range of frequencies (Figure 6). The expected location of maximum displacement for a given frequency is predicted by the Greenwood frequency-position function. While the model’s results closely align with Greenwood’s predictions, there are minor deviations in positioning. Despite these deviations, the model exhibits the same linear downward trend, indicating that it accurately simulates the key behaviors of the basilar membrane. On average, the model without a cochlear implant showed a 1.47 mm difference from the Greenwood predictions, while the implanted model showed a slightly larger average difference of 1.53 mm. The discrepancies are more pronounced at both very high and very low frequencies.

## 4. Discussion

The present study developed a finite element (FE) model of the inner ear from rhesus macaque MRI data, incorporating detailed anatomical structures. The model was validated through multiple simulations, including one with cochlear implant electrodes, demonstrating its potential for future applications in auditory research.

The results from the CI simulation in this study show that the insertion of a new cochlear implant design into the scala tympani minimally affects residual hearing in rhesus macaques. Changes in basilar membrane displacement, particularly at lower frequencies, were noted post-implantation, which might correspond to a slightly altered perception of sound, potentially “muddier” in real-world scenarios. Importantly, the model indicated that the location and amplitude of maximal displacement remained consistent between the unimplanted and implanted scenarios. This suggests that the basilar membrane’s tuning ability is preserved, allowing accurate pitch and volume perception despite the presence of the implant. Consequently, these findings suggest that any residual hearing loss post-implantation is likely not due to the implant itself.

Instead, the results point to other factors, such as surgical trauma or post-surgical scarring, as the main contributors to residual hearing loss. This is supported by the simulation of a stiffened round window membrane (RWM), mimicking post-implantation scarring. In this scenario, while the location of maximal displacement remained unchanged, the amplitude became distorted. These findings align with prior studies with the chinchilla ear [16] and the human ear [6], emphasizing that minimizing post-implantation scarring is critical for preserving hearing function. Therefore, future electrode designs must prioritize minimizing tissue damage during insertion and reducing scarring post-surgery.

This study not only reinforces existing knowledge on cochlear implant mechanics but also serves as proof of concept for utilizing a rhesus macaque FE model. The model offers a cost-effective and efficient platform for testing cochlear implant designs before moving to in vivo studies. Moreover, its potential extends beyond cochlear implants. Future iterations of the model could investigate other aspects of cochlear mechanics, such as how changes in labyrinth tissue stiffness or alterations in fluid dynamics within the vestibular system influence hearing and balance.

Additionally, the model holds promise for electrical simulations, particularly when combined with nerve tissue from previous µMRI scans and Ansys’s electrical modeling capabilities. This would allow for simulations of current spread from cochlear or vestibular implants, offering a foundation for optimizing electrode design for improved efficiency.

This study lays the groundwork for further investigations into cochlear implant design and function, leveraging the rhesus macaque model for both physiological and electrical simulations. Over the coming years, we plan to extend the model’s utility by incorporating simulations for eVOR/ECAP assessments, enhancing its role in pre-surgical evaluations and prognosis predictions.

## Figures and Tables

**Table 1:** Harmonic response simulation parameters applied to the stapes to mimic sound wave transmission through the ossicles and into the ear’s endolymph.

Frequency (Hz)	Amplitude (nm)	Phase (°)
1000	8.00	−300
2000	2.00	−380
4000	0.35	−470
6000	0.10	−510
8000	0.09	−585
10,000	0.05	−650

**Table 2:** Root mean square error between basilar membrane displacement in the unimplanted model, implanted model, and implanted model with stiffened round window membrane.

Frequency (Hz)	RMSE: Unimplanted vs. Implanted	RMSE: Unimplanted vs. Implanted + Stiffened RWM
1000	0.19066	0.35077
2000	0.08025	0.09430
4000	0.00534	0.01260
6000	0.00407	0.00851
8000	0.00270	0.00397
10,000	0.00107	0.00202

## Data Availability

The data presented in this study are available on request from the corresponding author. The data are not publicly available due to the policy of the University of Oklahoma.

## Abbreviations

CI: Cochlear implant; FE: Finite element; CA: Crista ampullaris, UC: Utricle, SA: Striola, MC: Macula, SC: Saccule, OWM: Oval window membrane, RM: Reissner’s Membrane, SV: Scala vascularis, SM: Scala media, ST: Scala tympani, RWM: Round window membrane; SSCs: Semicircular canals. MCA: Mesh convergence analysis.

## Appendix A

**Table A1:** Material properties of the model.

Mechanical Properties		
Structure	Value	Source
Basilar Membrane		
Density (kg/m^3^)	1.20 × 10^3^	[28]
Elastic Modulus (Pa)	$10^{5.7}\times e^{-0.19\times x}$	Experimentally Determined
ß Damping Coefficient	$10^{-5.91}\times e^{0.1\times x}$	Experimentally Determined
Poisson’s Ratio	0.4	Experimentally Determined
Reissner’s Membrane		
Density (kg/m^3^)	1.20 × 10^3^	[28]
Elastic Modulus (Pa)	1.20 × 10^3^	Experimentally Determined
ß Damping Coefficient	5.00 × 10^−3^	Experimentally Determined
Poisson’s Ratio	0.4	Experimentally Determined
Cupula		
Density (kg/m^3^)	1.20 × 10^3^	[40]
Elastic Modulus (Pa)	5.4	[41]
ß Damping Coefficient	5.00 × 10^−5^	[42]
Poisson’s Ratio	0.48	[40]
Maculae		
Gel layer		
Density (kg/m^3^)	1.00 × 10^3^	[43]
Elastic Modulus (Pa)	10	[43]
ß Damping Coefficient	0.01	[43]
Poisson’s Ratio	0.49	[43]
Otoconial layer		
Density (kg/m^3^)	2.00 × 10^3^	[43]
Elastic Modulus (Pa)	200	[43]
ß Damping Coefficient	0.01	[43]
Poisson’s Ratio	0.49	[43]
Round Window Membrane		
Density (kg/m^3^)	1.20 × 10^3^	[28]
Elastic Modulus (Pa)	3.50 × 10^5^	[28]
ß Damping Coefficient	5.00 × 10^−5^	[17]
Poisson’s Ratio	0.49	[44]
Oval Window Membrane		
Density (kg/m^3^)	1.20 × 10^3^	Assumed same as RWM
Elastic Modulus (Pa)	3.50 × 10^5^	Assumed same as RWM
ß Damping Coefficient	5.00 × 10^−5^	Assumed same as RWM
Poisson’s Ratio	0.49	Assumed same as RWM
Annular Stapedial Ligament		
Density (kg/m^3^)	1.20 × 10^3^	[42]
Elastic Modulus (Pa)	2.00 × 10^5^	[32]
ß Damping Coefficient	5.00 × 10^−5^	[42]
Poisson’s Ratio	0.3	[28]
Osseus Spiral Lamina		
Density (kg/m^3^)	1.20 × 10^3^	[30]
Elastic Modulus (Pa)	1.41 × 10^10^	[30]
ß Damping Coefficient	1.00 × 10^−4^	[42]
Poisson’s Ratio	0.3	[30]
Membranous Labyrinth		
Density (kg/m^3^)	1.20 × 10^3^	[42]
Elastic Modulus (Pa)	1.30 × 10^4^	Experimentally Determined
ß Damping Coefficient	0.14	Experimentally Determined
Poisson’s Ratio	0.4	[21]
Lymphatic Fluids		
Density (kg/m^3^)	1.00 × 10^3^	[40]
Bulk Modulus (Pa)	2.20 × 10^9^	[42]
Poisson’s Ratio	0.49	Assumed
Viscosity (Pa×s)	8.50 × 10^−3^	[40]
Damping Coeff. β (s)	1.00 × 10^−4^	[17]
Cochlea Electrode		
Density (kg/m^3^)	1.13 × 10^3^	[33]
Elastic Modulus (Pa)	1.65 × 10^6^	[33]
ß Damping Coefficient	0.0414	[33]
Poisson’s Ratio	0.499	[33]
